# Sensitivity and Specificity of Ultrasonography Using Ovarian-Adnexal Reporting and Data System Classification Versus Pathology Findings for Ovarian Cancer

**DOI:** 10.7759/cureus.17646

**Published:** 2021-09-01

**Authors:** Dania Guadalupe Solis Cano, Hugo Alberto Cervantes Flores, Omar De los Santos Farrera, Nancy Berenice Guzman Martinez, Danny Soria Céspedes

**Affiliations:** 1 Radiology, Centro Médico ABC, The American British Cowdray Medical Center, Ciudad de México, MEX; 2 Health Sciences/Medical Sciences, Universidad Anáhuac México, Ciudad de México, MEX; 3 Pathology, Centro Médico ABC, The American British Cowdray Medical Center, Ciudad de México, MEX; 4 Pathology, Centro Médico ABC, The American British Cowdray, Ciudad de México, MEX

**Keywords:** adnexal mass, sensitivity, specificity, o-rads, pelvic ultrasound

## Abstract

Background

One of the limiting factors for early diagnosis of ovarian neoplasms is the lack of standardized terminology for ultrasound. The Ovarian-Adnexal Reporting and Data System (O-RADS) classification aimed to reduce variability between observers and facilitate communication with attending physicians. Recent studies show that O-RADS has higher sensitivity (96.8%) and specificity (92.8%) compared to other classifications. However, to date, there are no reports on O-RADS correlation with pathology findings.

Objectives

To determine sensitivity and specificity of ultrasound, as a tool for detecting malignant ovarian neoplasms, using the O-RADS compared to pathology reports.

Materials and methods

We evaluated 73 transvaginal ultrasound records with adnexal masses and applied the O-RADS system. Then, we compared against definitive histopathology diagnosis. We calculated sensitivity and specificity using SPSS.

Results

O-RADS sensitivity for detection of ovarian cancer was 52%, with a specificity of 84%, negative predictive value of 79%, and positive predictive value of 60%, with an accuracy of 73%.

Conclusions

In our study, O-RADS classification yielded a higher specificity than sensitivity for malignant vs. benign findings. Hence, we propose that this classification could be useful for tailoring treatment appropriately. O-RADS 0 to 2 may benefit from conservative treatment while O-RADS 3 to 5 may require surgical treatment.

## Introduction

According to GLOBOCAN (2020), the standardized incidence rate for ovarian cancer in all ages is 6.6, with a mortality rate of 4.2 to 5 years per 100,000 inhabitants. The incidence rate for ovarian cancer in women of all ages was 313,959 new cases with 207,252 deaths and a rate of 21.3 per 100,000 inhabitants. Ovarian cancer was ranked 19th in incidence and 15th in mortality among all cancers for both sexes.

In Mexico, the reported incidence rate was 4,963 at five years and mortality was 3,038 deaths, making ovarian cancer ranked as the 14th in incidence and 12th in mortality. All-age prevalence was reported to be 13,529 cases per 100,000 inhabitants at five years. Ovarian cancer is most prevalent in postmenopausal women, with peak incidence rates at 50 to 75 years old (median age is 63) [1].

In August 2020, the National Institute of Cancerology (Instituto Nacional de Cancerología, INCan) and the National Institute of Public Health (Instituto Nacional de Salud Pública, INSP) report, placed ovarian cancer third in prevalence among all gynecological cancers. 95% were epithelial variants and the remaining 5% corresponding to germ cell cancers. Worldwide, epithelial variants remain the main cause of mortality because most patients (70%-80%) are diagnosed at advanced stages. This could be explained by a lack of symptoms during early disease, as well as inadequate or nonexistent screening techniques [2].

Although no specific tests exist for ovarian cancer screening, ultrasound evaluation can adequately characterize most uterine adnexal masses, both benign and malignant. Its low cost and lack of risk associated with ionization radiation, are also advantageous for use as a screening tool [3].

The RADS classifications, mainly oriented towards cancer-related imaging, include standardized terminology and are evaluated and certified by the American College of Radiology (ACR). Their main objectives are reducing variability between reports and facilitating communication between radiologists and attending physicians.

First proposed in 2018 by the ACR, the Ovarian-Adnexal Reporting and Data System (O-RADS) is a method for categorizing ovarian masses, mainly by ultrasound [4]. Although systems such as the International Ovarian Tumor Analysis (IOTA) and GI-RADS have already targeted this goal, recent studies show that O-RADS has higher sensitivity (96.8%) and specificity (92.8%) [4,5].

Based on this, and correlating with histological findings, we aim to provide more conservative measures for the treatment of benign adnexal masses, as well as treating early-onset disease [6,7].

## Materials and methods

A retrospective study was made in Centro Médico ABC Hospital, a tertiary care center in Mexico City. Ultrasound reports and medical records from all patients from 2017 to 2021 were evaluated. All ultrasounds were performed in a standardized equipment Hitachi model EUP-C532 (8-4) series KE11193302F by two highly experienced ultrasonography technicians. All images were reviewed by an expert radiologist blinded to the final pathology report. Inclusion criteria were: adult females, with suspected or confirmed ovarian neoplasms. Exclusion criteria were nubile patients and those with previous uterine adnexal surgery.

**Figure 1:**
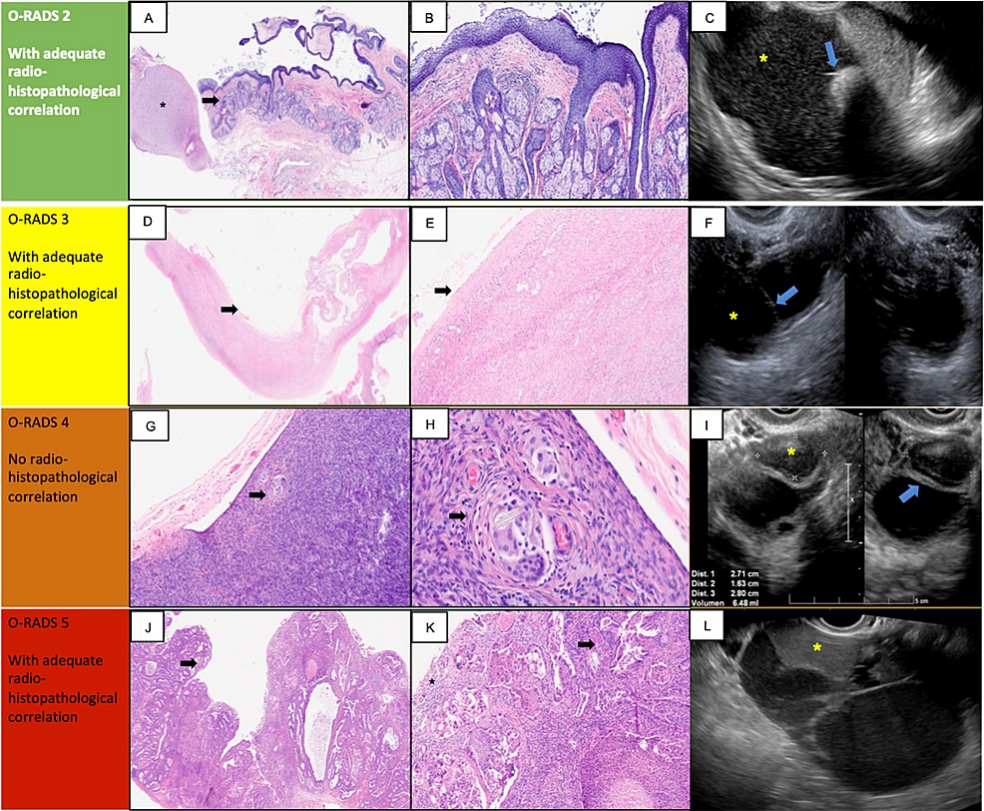
Representative ultrasound images and histopathological correlation. A-B. Microscopic images of the cystic lesion wall showing brain tissue (asterisk) and skin (arrow) composed of epidermis, hair follicles and sebaceous glands corresponding to mature cystic teratoma (A. 20x H and E, B. 100x H and E). C.Transvaginal ultrasound image shows a lesion with a hyperechoic component casting an acoustic shadow ( blue arrow)  and scattered small high density, nonvascular foci (yellow asterisk). D-E. Microscopic images of the wall of a cystic lesion with dense fibrosis, cellular ovarian stroma and attenuation of the lining epithelium (arrows) corresponding to serous cystadenoma. (C. 20x H and E, D. 100x H and E). F. Transvaginal ultrasound shows a cystic lesion (yellow asterisk) and thin internal septation (blue arrow), with an axis> 10 cm. G-H. Microscopic images of the ovarian cortex with foreign-body type multinucleated giant cells, in whose cytoplasm show phagocytosed refringent material (arrows) corresponding to a foreign-body type granulomatous reaction (G. 40x H and E, H. 400x H and E). I.Transvaginal ultrasound image shows a multilocular cystic with a solid component (yellow asterisk)  and internal wall thickened (blue arrow). J-K. Microscopic images of a malignant neoplasm composed of endometrioid carcinoma (arrows) and high-grade serous carcinoma (asterisk), corresponding to mixed ovarian carcinoma (I. 20x H and E, J. 100x H and E). L. Transvaginal ultrasound image showing a multilocular cyst with a solid component (yellow asterisk).

Transvaginal ultrasound reports were classified, according to O-RADS, as benign findings (O-RADS 0-2) and suggestive of malignancy (O-RADS3-5). These diagnoses were compared with histopathology reports made by two experienced pathologists, then these reports were divided into benign or malignant findings, excluding immature teratoma, serous cystadenoma, and mixed carcinoma (Figure 1). There was an adequate histopathological correlation between pathology slides and ultrasound images for O-RADS classifications 2,3,5, as depicted in Figure 1.

The study was authorized by the ethics committee at our institution, ABC 20-94 approval number.

The sample size was calculated (n=73) with a confidence interval of 97%.

Data analysis was performed using SPSS (IBM Corp. Released 2013. IBM SPSS Statistics for Windows, Version 22.0. Armonk, NY: IBM Corp). Continuous variables with normal distribution were presented with median and standard distribution. 2X2 contingency tables were used for sensitivity and specificity calculation. P < 0.05 was considered statistically significant.

## Results

Data from 73 female adult patients with a median age of 42±11 years of age were evaluated without and with symptoms such as abdominal pain, palpable mass, abnormal uterine bleeding, anorexia, weight loss, asthenia, constipation, amenorrhea, referred by their physician (Table 1).

**Table 1:** Sample description.

Variables	Total	%
Females aged 18 years or older.	73	100%
Symptoms		
Yes	52	71.2%
No	21	28.8%

Using the ultrasound reports obtained from the PACS (picture archiving and communication system) with prior approval from ABC Medical Center's ethics committee (folio ABC-20-94) and classifying them by O-RADS, 72.6% corresponded to benign findings and 27.4% to malignant ones. The histological reports provided by the pathology service were 68.5% benign and 31.5% premalignant or malignant (Table 2).

**Table 2:** Findings in O-RADS and histopathology reports. O-RADS: Ovarian-Adnexal Reporting and Data System.

Variables	Frequency	Percentage
Ultrasound reports (O-RADS)		
Benign (ORADS 0-2)	53	72.6%
Malignant (ORADS 3 -5)	20	27.4%
Histopathology (diagnoses)		
Benign	50	68.5%
Malignant and pre-malignant	23	31.5%

O-RADS sensitivity for detection of ovarian cancer was 52%, with a specificity of 84%, a negative predictive value of 79%, and a positive predictive value of 60%, with an accuracy of 73% (Table 3).

**Table 3:** Cross table. O-RADS: Ovarian-Adnexal Reporting and Data System.

		Histopathology		
		Malignant and pre-malignant	Benign	Total
Ultrasound	O-RADS 3-5	12	8	20
	O-RADS 0-2	11	42	53
Total		23	50	73

## Discussion

Pelvic ultrasound is still the best imaging method for the evaluation of adnexal masses, allowing the operator to classify tissue according to grayscales as well as colored Doppler mode [8].

Surgical excision is currently the most commonly accepted management for adnexal masses. However, most cystic masses are benign and can be adequately characterized and followed with ultrasound without surgical treatment. Although the consensus of the Society of Radiologists in Ultrasound concurs, the lack of standardized terminology precludes the usage of their criteria for determining high-risk cystic lesions. Thus, the limiting factor for early diagnosis of ovarian tumors and their prompt treatment is the lack of standardized procedures and terminology for a pelvic ultrasound [9,10].

Previous studies have correlated demographics, pre-operative ultrasound findings, and CA-125 levels with the malignancy index score and the IOTA terminology in order to predict the post-operative and histopathology definitive diagnoses. Ultrasound studies using IOTA terminology showed 83.8% sensitivity and 92.0% specificity, significantly higher than the malignancy index score, with 77.2-82.1% sensitivity and 82.6%-86.8% specificity [9-11]. In our study based on O-RADS classification, we measured sensitivity for detection of ovarian cancer is 52%, a specificity of 84%, a negative predictive value of 79%, and a positive predictive value of 60%, with an accuracy of 73%. However, more evidence is needed to fully ascertain the diagnostic accuracy among other ethnicities/populations.

The current trend is seeking to minimize unnecessary surgical procedures on low-malignancy cases, thus minimizing surgical morbidity and preserving ovarian function in patients with low malignancy risk. The need for universally accepted terminology in ultrasound reporting for ovarian and adnexal masses is essential for this goal. O-RADS provides standardized terminology that includes all necessary descriptors and definitions for adequately characterizing normal ovaries and ovarian or adnexal tumors. The O-RADS working group defined six categories for classifying malignancy risk: O-RADS 0 for unsatisfactory or inconclusive data, O-RADS 1 for normal pre-menopausal ovaries, O-RADS 2 for benign findings (<1% malignancy risk), O-RADS 3 for low malignancy risk (1%-10%), O-RADS 4 for intermediate-risk (10%-50%), and O-RADS 5 for high malignancy risk (≥50%) [3]. Overall, patients with an O-RADS score of 0-2 will have a 79% probability of having a benign tumor, while those with O-RADS 3-5 will have a 60% probability of having a malignant tumor. Interestingly, our study showed that O-RADS was more specific for malignancy detection. More studies are needed to fully elucidate this finding.

## Conclusions

In our study, O-RADS classification yielded higher sensitivity than specificity for malignant vs. benign findings. Hence, we propose that this classification could be useful for tailoring treatment appropriately. O-RADS 0 to 2 may benefit from conservative treatment while O-RADS 3 to 5 may require surgical treatment. However, more studies are needed, especially in other populations/ethnicities, to fully ascertain the role of ultrasonography using the O-RADS classification for ovarian benign or malignant disease.
